# Prognostic factors in oral and oropharyngeal cancer based on ultrastructural analysis and DNA methylation of the tumor and surgical margin

**DOI:** 10.1007/s13277-014-1958-1

**Published:** 2014-05-01

**Authors:** Daniela Mielcarek-Kuchta, Jarosław Paluszczak, Monika Seget, Katarzyna Kiwerska, Wiesława Biczysko, Krzysztof Szyfter, Witold Szyfter

**Affiliations:** 1 Department of Otolaryngology and Clinical Oncology, University of Medical Sciences, ul. Przybyszewskiego 49, 60-355 Poznań, Poland; 2 Department of Pharmaceutical Biochemistry, University of Medical Sciences, Poznań, Poland; 3 Department of Clinical Pathology, University of Medical Sciences, Poznań, Poland; 4 Institute of Human Genetics, Polish Academy of Sciences, Poznań, Poland

**Keywords:** Oral cancer, Ultrastructural examination, Methylation, Prognostic factors

## Abstract

Oral and oropharyngeal cancers are characterized by relatively low 5- year survival rates due to many factors, including local recurrence. The identification of new molecular markers may serve for the estimation of prognosis and thus augment treatment decisions and affect therapy outcome. The aim of this study was to describe the morphological characteristics and the DNA methylation status of the CDKN2A,CDH1, ATM, FHIT and RAR- genes in the central and peripheral part of the tumor and the surgical margin and evaluate their prognostic significance. 53 patients with oral and oropharyngeal cancer were enrolled to the prospective study, and had been primarily treated surgically. Correlations between morphological data, hypermethylation status and clinicopathological data, as well as prognosis, were assessed. Nuclei polymorphism highly correlated with T stage (*p* < 0.0001), N stage (*p* < 0.046), and metastases to the lymph nodes pN (*p* < 0.004 ). Also, the number of cells in irregular mitosis correlated with T stage (*p* < 0.004), and highly with pN (*p* < 0.009). The significance of CDKN2A hypermethylation as a good prognostic factor was also established in the Kaplan-Meir test. The ultrastructural analysis showed that none of the examined tumors had homogenous texture and that resection margin specimens clean in HE stained tissue samples frequently contained single tumor cells or few cells in groups surrounded by connective tissue. This indicates the superiority of electron microscopy over standard histopathological analysis. Thus, a combination of such morphological examination with epigenetic parameters described herein could result in the discovery of promising new prognostic markers of the disease.

## Introduction

Oral cancer is a serious and growing medical problem. It is the sixth most common cancer in the world and occurs in people aged 50 and over [1, 2]. However, about 6 % of patients suffering from this type of disease are under 45 years old, or even under 40 in countries with a high incidence [3–6]. The clinical data regarding the course of the disease are not clear-cut [7]. The risk factors are well known (tobacco smoking, alcohol abusing), but there are currently many reports regarding non-smoking and non-drinking populations with HPV infection [8–11]. Treatment failure is still high; thus, the search for prognostic markers which could be useful in early detection of recurrence is ongoing.

Among many prognostic factors, the assessment of surgical margin seems to be crucial. Routine histopathological examination using hematoxylin–eosin staining of paraffin-embedded tissue samples is a golden standard in evaluation of the resection field by pathologists. However, this technique does not allow to describe the ultrastructure of the tumor and surgical margin. For the last-mentioned purpose, the epoxy resin-embedded samples are prepared. Then, before electron microscopy studies are done toluidine blue-stained semi-thin sections are examined. Using this technique, the pathologist may evaluate more details including nuclear polymorphism, cells in mitosis, as well the cellular characteristic (size, shapes, size of nuclei, number of nucleoli per nucleus, presence of polykaryocytes, typical cytoplasmic ultrastructural characteristic for squamous epithelium). This technique allows also to find more precisely suspected changes, as dysplastic or neoplastic cell “traveling” through normal tissue. Besides this, immunohistochemistry and molecular analysis could bring new information. It has to be underlined that genetic and epigenetic changes are also detected in histopathologically clean resection fields. It could cause local relapse in mucosa primarily free of cancer cells. This has been explained by the Slaughter’s model of “field cancerization” [12]. In turn, the Califano model is based on the increased number of genetic alterations in the field, with a division into early and late ones [13]. Finally, there is the “patch-field” model proposed by Braakhuis [14]. All these theories assume that the whole mucosa is predisposed to carcinogenesis due to exposure to exogenous genotoxins [15, 16].

Among the molecular alterations found in the early stage of carcinogenesis are epigenetic changes. Silencing of tumor suppressor genes associated with promoter hypermethylation is a common feature in human cancers and serves as a mechanism for the loss of their function. [17–19]. The hypermethylation status of different genes was stated to be important for prognosis in cancer patients. Methylation analyses are performed frequently using tumor tissue, but some results showed that the estimation of DNA methylation changes in the resection margin has an even greater prognostic value [20].

The epigenetic alterations of *CDKN2A* (*p16*; located at 9p21), *CDH1* (16q22.1), *FHIT* (3p14.2), *RAR-*β (3p24.2), and *ATM* (11q22.3) have been found to be related to clinical prognosis in cancer of the head and neck [18]. The analysis of methylation of *CDKN2A* gene has been suggested to be a useful method in the molecular diagnostics of the resection field [21]. The hypermethylation of *CDH1* gene was considered to be a bad prognostic factor in tongue cancer [22] and *ATM* gene hypermethylation was connected with poorer prognosis in head and neck cancers [23]. Methylated *RAR-*β gene is completely suppressed in immortal dysplasia and oral carcinoma [24]. However, the results are still inconsistent; thus, the search for epigenetic markers useful as prognostic factors remains still an open question.

The aim of this study was to establish the morphological characteristics of various parts of the tumor including comparisons between the central, peripheral part and the surgical margin of the cancer of the oral cavity and oropharynx. We also assessed the methylation status of five genes (*CDKN2A*, *CDH1*, *ATM*, *FHIT*, and *RAR-*β) in the same samples. In the following study, the correlations between morphological data, genes hypermethylation status, clinicopathological data, and survival were assessed.

## Material and methods

### Patients

Patients with oral and oropharyngeal cancer who had been primarily treated surgically in the Department of Otolaryngology and Clinical Oncology, University of Medical Sciences in Poznań, Poland, between July 2009 and April 2012 were enrolled to the prospective study. The study group consisted of 53 patients, 4 women and 49 men, aged 24–82. The clinical data including sex, age, stage of the tumor, histological grading, risk factors, and localization of the tumor are listed in Table 1. The tissue samples were taken from the central (A) and peripheral part of the tumor (B) and from the surgical margin (C). All samples underwent routine histopathological examination (HE-stained specimens) and ultrastructural assessment using toluidine blue staining in a light microscope and in transmission electron microscopy (EM). The resection margin was considered clean in histopathological examination, if the distance from the invasive cancer to the margin was more than 5 mm—according to the UK Royal College of Pathologists. DNA methylation status for the chosen genes was established in the same samples (A, B, and C).

**Table 1:** The clinical characterization of the study group

Number of patients	53
Male	49
Female	4
Age (years)	
Range	29–82
Mean	57
Habits	
Smoking	35
Smoking and alcohol	13
No drinking and no smoking	5
TNM	
T1	6
T2	24
T3	12
T4	11
N0	25
N1	15
N2	12
N3	1
M0	0
G stage	
G1	7
G2	41
G3	5
Tumor localization	
Tonsils (no infiltration of the base of the tongue)	13
Tongue (2/3 anterior, no infiltration of the floor of the mouth)	16
Tongue and floor of the mouth	11
Palate	3
Lips	1
Base on the tongue and tonsils	8
Cheek	1

### Ultrastructural assessment

Tissue samples were pre-fixed in Karnovsky solution (glutaraldehyde 2 % and paraformaldehyde 3.4 % in 0.1 M cacodylate buffer pH 7.4) overnight (1 h at room temperature and cooled down to 4 °C). After pre-fixation, samples were washed several times in 0.1 M phosphate buffer and post-fixed in 1 % OsO_4_ in 0.1 M phosphate buffer with K_4_Fe(CN)_6_ · 3H_2_O; 0.16 % for 3 h at +4 °C. After post-fixation, dehydration in gradually increasing concentrations of ethanol and then acetone was carried out. Embedding in Epon 812 and polymerization were the next steps of the procedure. Semi-thin sections were cut from all blocks, stained with toluidine blue of basic pH, and checked with the use of an Olympus BX 41. After selection of the proper slide area ultrathin sections were cut and doubly stained with uranium acetate and lead citrate for EM. Selection of ultrathin section areas and micrographs was done by JEM 1011.

Since the aims of the study also included a careful check of the margins of tumors and their periphery (oncologic sterility), the control performed on the base of semi-thin sections was very accurate. In case of any doubts concerning presence of single tumor cells in surrounding tissue, EM checks were done.

In this series, we compared groups with a clean resection margin in HE-stained specimens and a positive margin in morphological assessment, versus a group with a clean resection margin in both examinations with survival, TNM status, G stage, and pN.

### DNA extraction and gene methylation analysis

DNA was isolated from all samples according to standard procedures (proteinase K digestion, phenol/chloroform extraction, and ethanol precipitation). For DNA methylation analysis, samples with more than 80 % of cancer cells in the field were enrolled. The methylation status of *RAR-*β, *CDH1*, *FHIT*, *CDKN2A*, and *ATM* was assessed using the methylation-specific polymerase chain reaction (MSP). DNA was converted in the presence of sodium bisulfite using the EZ DNA Methylation Kit from ZymoResearch (Orange, CA, USA). TrueStart Hot Start Taq DNA Polymerase from Fermentas (Burlington, Canada) was used for the amplification of *RAR-*β and *ATM*, whereas FastStart Taq DNA Polymerase (Roche Diagnostics, Germany) was used for the amplification of *CDH1*, *FHIT*, and *CDKN2A*. The primers and reaction conditions for MSP were as previously reported in other studies [25–29]. All the primers were obtained from Oligo.pl (Warsaw, Poland). DNA extracted from the lymphocytes of healthy blood donors was used as a negative control, and completely methylated human DNA (New England Biolabs, Ipswich, MA, USA) was used as a positive control in the MSP reactions. Amplification products were separated on 2.5 % agarose gels and visualized in UV light after ethidium bromide staining.

First, we compared the results obtained for groups A, B, and C: samples with methylation vs those without it in relation to TNM, pN, G stage, and survival. Then, groups A and B were coupled (tumor zone) and these results were correlated with clinicopathological data.

### Statistical analysis

The correlation between clinicopathological features and results of morphological examination, as well as gene methylation, was assessed using Chi-square, Mann–Whitney, and Kaplan–Meier tests (*p* < 0.05). The Statistica program was used for analysis.

## Results

**Fig. 1:**
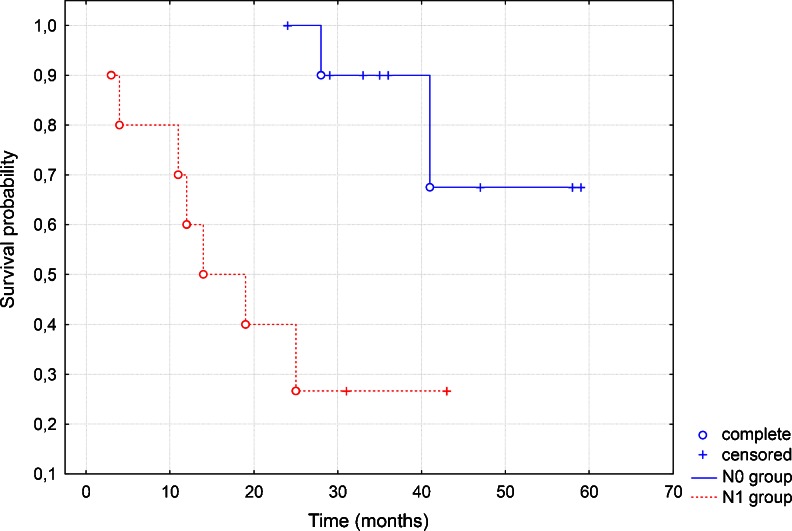
The chart is showing differences between two groups: without neck metastases (pN0) and clean margin, and with neck metastases (pN1) and positive margin in HE staining. The first one presents longer survival in the Kaplan–Meier test

In the study group, the T2 stage of the tumor, N0 nodal involvement, and G2 malignancy grading prevailed. Of the 53 patients, 12 had a positive surgical margin in histopathological examination. In the series with a clean resection margin, we did not find histopathologically confirmed node metastases (pN) and the result was statistically significant (*p* = 0.0393). The second very important point was the finding that the group with pN0 and clean margin in HE staining, but having cancer cells in toluidine blue staining, present longer survival in the Kaplan–Meier test (Fig. 1).

**Fig. 2:**
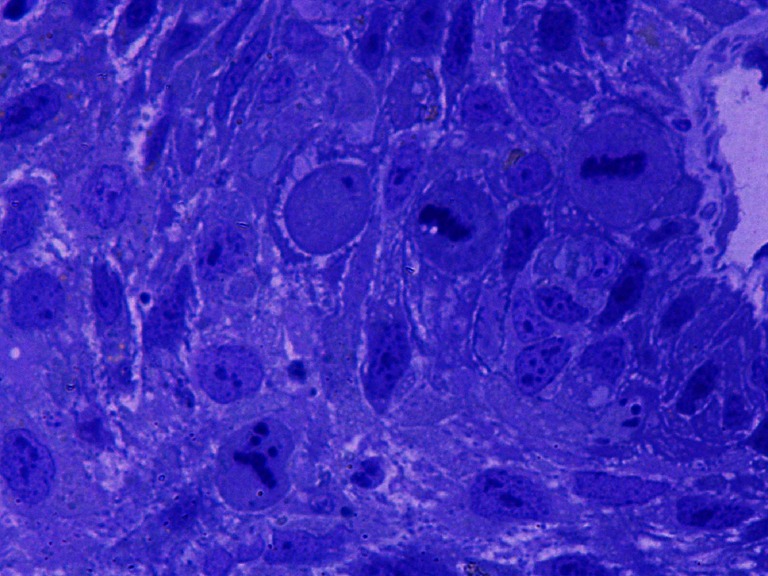
The picture shows non-homogenous structure of the tumor

**Fig. 3:**
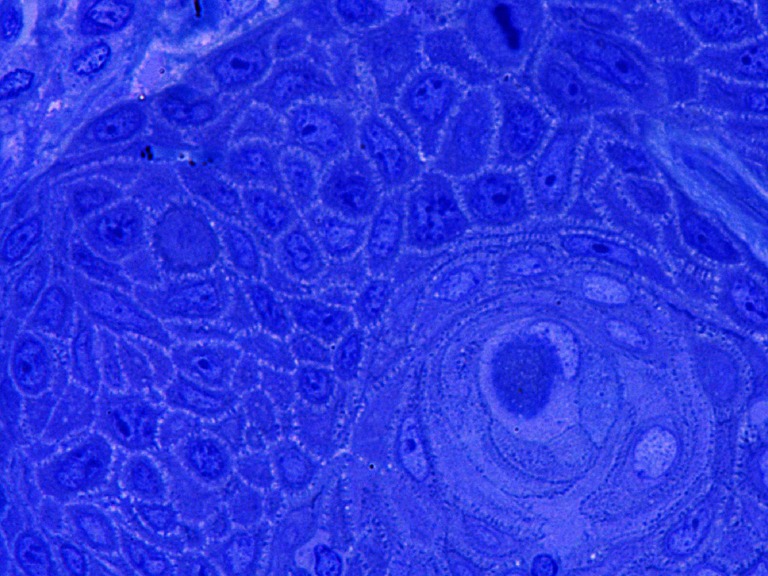
This figure shows horny pearl in G1 texture; other cells are modestly monomorphic in G1-2 carcinoma

**Fig. 4:**
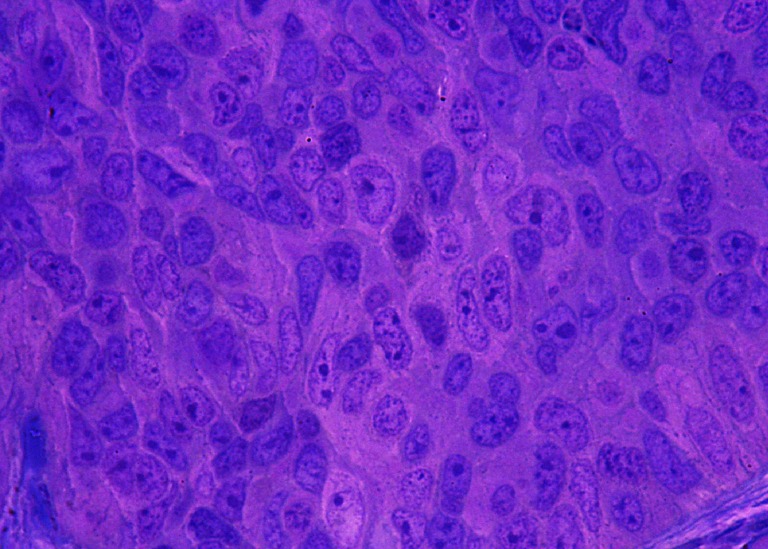
Solid area of the G2-G3 stage of the tumor. Significant polymorphism of nuclei with numerous nucleoli dominates in the general picture of this tumor. The area occupied by nuclei evidently prevail over the area of cytoplasm

**Fig. 5:**
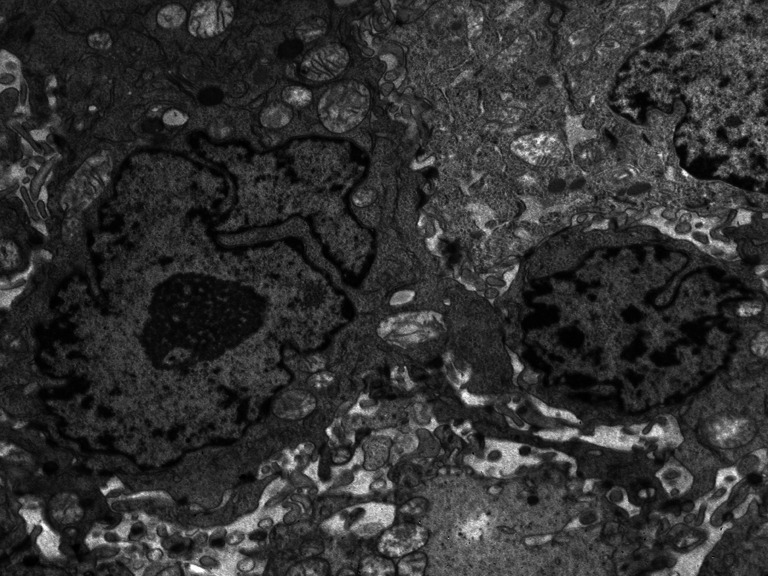
The micrograph presents differences between the nuclei of tumor cells. One is much larger, with a deeply invaginated nuclear membrane, enlarged active nucleolus, a field of interchromatin granules, and small groups of heterochromatin located in the vicinity of the nuclear membrane

**Fig. 6:**
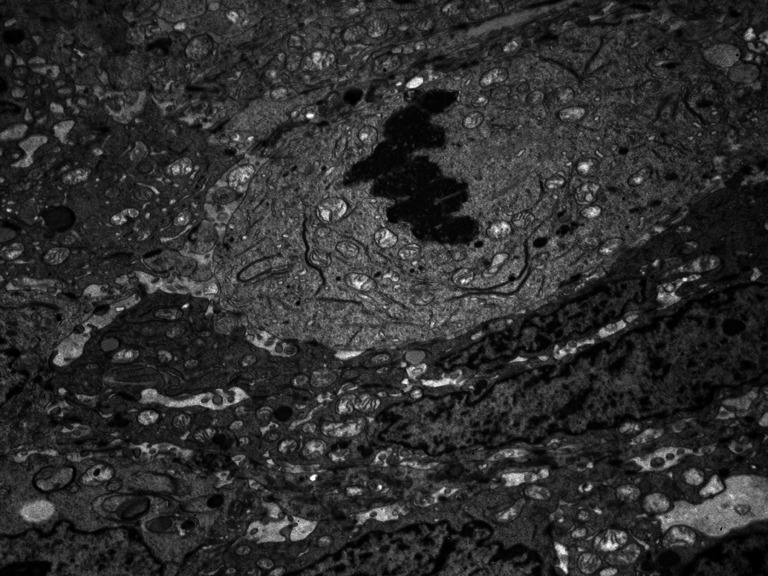
One of the tumor cells forms chromosomes and removes the nuclear membrane. Display of organelles is typical for the prophase. Neighboring cells do not divide

**Fig. 7:**
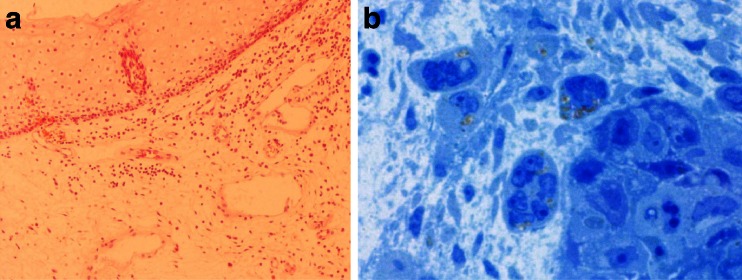
Comparison between HE- (**a**) and toluidine blue- (**b**) stained specimens of the same patient. First one were classified as a clean resection margin; however, in the second one single cancer cells are present

None of the tumors collected for research had a homogeneous texture (Fig. 2). In all cancers, fields of differentiated cells maturating to fully keratinized—or in the form of keratinizing—horny pearls (Fig. 3), or small horny plates lying on the free surface of the tumor occurred. Apart from maturating cells, others which had cytoplasmic features typical for spinous cells, i.e., cytokeratin bunches and irregular cytoplasmic bridges with irregular desmosomes or other forms of intercellular junctions, were found. Cells differed in size and shape of nuclei and cytoplasm (Fig. 4). Nuclear polymorphism was distinct and considerable, especially in the invasive front of the tumor. The ultrastructural characteristics of nuclei were very different for these cells compared to the nuclei of maturating cells. They were large, with an irregular contour, often with deep invagination of the nuclear envelope into the nucleoplasm (Fig. 5). Many nuclei had large, active, and numerous nucleoli. Parts of the nuclei were euchromatinized and had active nucleoli and very numerous perichromatin granules, with different diameters and areas of interchromatin granules. This is typical for cells which enter into the cell cycle. In the whole collected material, cells occurred in mitosis—and some of them were atypical or endomitotic, what could be interpreted as a loss of proper cell cycle control (Fig. 6). In some of the assessed tumors, disorders of the late telophase were found as a partial loss of the nuclear envelope. All of the studies carried out show that cancer formed heterogenous cellular populations, with differences of texture classified as different histological gradings: G1 or G2 or G3. Most commonly, the cells with lower G were found in the invasive front of the tumor. There was a statistically significant correlation between nuclei polymorphism and T stage (*p* < 0.0001), N stage (*p* < 0.046), and metastases to the lymph nodes confirmed in histopathological examination—pN (*p* < 0.004). The number of cells in irregular mitosis correlated with T stage (*p* < 0.004), and highly with pN (*p* < 0.009). In the examined group, there were differences between the clean resection margin in HE-stained specimens and those assessed using toluidine blue (Table 2). In the specimens prepared for the assessment of the ultrastructure of the tumor, even under a light microscope, it was possible to find cancer cells between normal glands, skeletal muscle cells, and connective tissue stroma (Fig. 7).

**Table 2:** The number and percentage of patients with clean and positive resection margin in two types of examinations

Margin U	Margin HE 0	Margin HE 1	Total
0	20 (37.74 %)	4 (7.55 %)	24 (45.28 %)
1	21 (39.62 %)	8 (15.09 %)	29 (54.72 %)
Total	41 (77.36 %)	12 (22.64 %)	53 (100 %)

*U* ultrastructural, *HE* hematoxylin and eosin-stained, *0* clean, *1* involved

We also correlated the group with a clean resection margin in HE-stained specimens but having cancer cells in ultrastructural examination with clinicopathological features. This group did not present a poor prognosis and did not correlate with TNM status or G stage. Apart from ultrastructural analysis, the methylation status of selected tumor suppressors was also assessed.

The methylation was found most often for *CDH1* and *CDKN2A* genes in the A and B groups. However, there was no statistically significant correlation between gene hypermethylation and stage of the tumor, nodal involvement, and G stage, except for a correlation between *CDH1* in the B and N1 stage (*p* = 0.031). For the next two genes, *RAR* β and *FHIT*, hypermethylation was found less frequently and also did not correlate with clinicopathological data. We did not detect methylation of *ATM* gene in any of the samples under analysis. Gene methylation was also found in the clean resection margin, and the percentage ranged between 1.8 and 26.4 (Tables 3 and 4). Altogether, the molecular changes in the resection margin did not correlate with TNM, malignancy grading, or survival. Likewise, combining the results of groups A and B did not provide any correlation between hypermethylation and clinicopathological data.

**Table 3:** The presence of gene hypermethylation in the central (A) and peripheral parts (B) of the tumor, as well in the surgical margin (C)

	A	B	C
*CDH1*	20/53	24/53	14/53
	(37.7 %)	(45.3 %)	(26.4 %)
*p16*	14/53	18/53	10/53
	(26.4 %)	(33.9 %)	(18.8 %)
*RARbeta*	4/53	0	3/53
	(7.5 %)		(5.6 %)
*FHIT*	1/53	1/53	1/53
	(1.8 %)	(1.8 %)	(1.8 %)
*ATM*	0	0	0

**Table 4:** The analysis of gene methylation in clean and involve resection margin in two types of examinations: U—ultrastructural and HE—hematoxylin and eosin-stained, “−”—clean, “+”—involved; A—central part of tumor, B—peripheral part of tumor and C—resection margin

	*ATM*	*CDH1*	*CDKN2A*	*FHIT*	*RAR-*β
HE+	0	A:5 (9.43 %)	A:3 (5.66 %)	0	A:1 (1.88 %)
		B:5 (9.43 %)	B:5 (9.43 %)		B:0
		C:3 (5.66 %)	C:4 (7.54 %)		C:0
HE−	0	A:13 (24.52 %)	A:10 (18.86 %)	A:1 (1.88 %)	A:2 (3.77 %)
		B:16 (30.18 %)	B:12 (22.64 %)	B:1 (1.88 %)	B:0
		C:9 (16.98 %)	C:6 (11.32 %)	C:1 (1.88 %)	C:2 (3.77 %)
U+	0	A:15 (28.30 %)	A:5 (9.43 %)	A:1 (1.88 %)	A:3 (5.66 %)
		B:15 (28.30 %)	B:7 (13.20 %)	B:1 (1.88 %)	B:0
		C:10 (18.86 %)	C:6 (11.32 %)	C:1 (1.88 %)	C:1 (1.88 %)
U−	0	A:5 (9.43 %)	A:7 (13.20 %)	0	A:1 (1.88 %)
		B:7 (13.20 %)	B:9 (16.98 %)		B: 0
		C:3 (5.66 %)	C:3 (5.66 %)		C:1 (1.88 %)

**Fig. 8:**
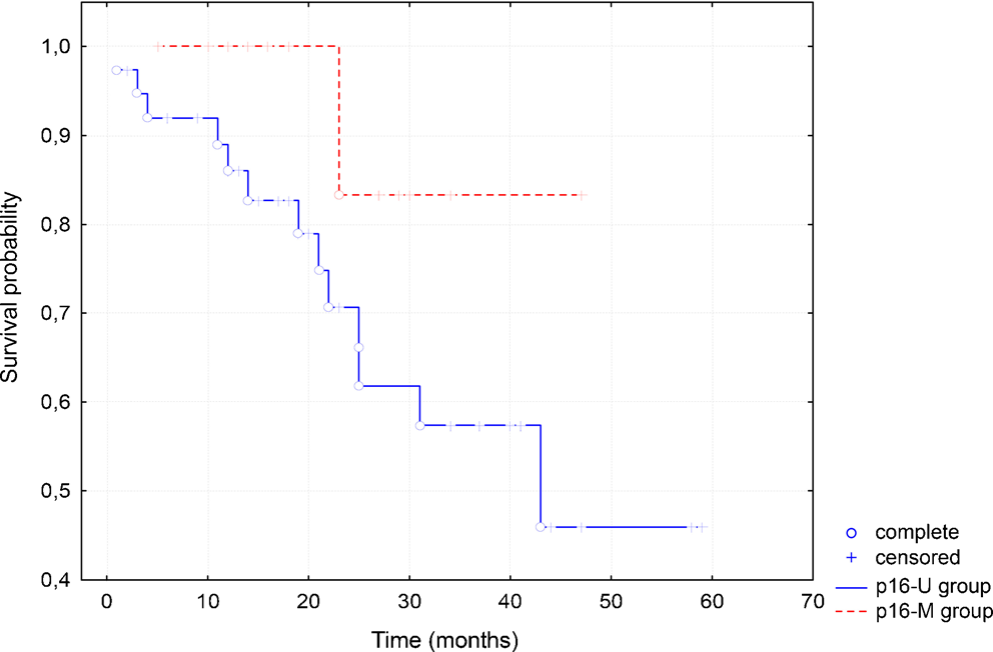
The chart shows that the group with p16 methylation (p16-M) presents better survival than the group without hypermethylation (p16-U) in the Kaplan–Meier test

In the Kaplan–Meier test, there was no statistically significant correlation between gene methylation and survival, except methylation of *CDKN2A*. The group with *CDKN2A* hypermethylation presented longer survival (Fig. 8).

## Discussion

Five-year survival rates in oral cancer are still unsatisfactory. The epidemiological data show some differences between Western and Eastern European countries, as well as the USA. However, they never cross the 50 % threshold [1, 7] that points at a deep need to find new prognostic factors.

Carcinogenesis is a multistep process [15, 16]. Cancer development is the result of the accumulation of molecular alterations. Epigenetic and genetic changes lead to the deregulation of cell cycle through (epi)mutations of tumor suppressor genes and proto-oncogenes. In some of the cells, mutated genes conform with a proliferative activity and are responsible for the progression of cells to invasive carcinoma. Environmental carcinogens can initiate this process in the area of epithelial cells. The same carcinogens have often a global effect on the whole oral epithelium. This fact can be responsible for the multifocal progression of mutated cells toward invasive carcinoma by the same oral mucosa and thus can be represented by the morphological heterogeneity of cancer texture in the analyzed material. Our study shows that none of the examined tumors had the homogenous structure. This tissue texture characteristic was represented in small, medium, and large tumors, and was not dependent on tumor primary location. The observations gathered in our study are in line with the ideas formulated by Braakhuis [14]. It can also be thought that progression of certain tumor cells to the invasive stage does not take place simultaneously [30].

The gold standard in the assessment of cancer tissue is histological examination using HE-stained samples. Evaluation of the same tissue using a transmission electron microscope improves the accuracy of the morphological characteristics of examined cells. In papers describing carcinomas of oral mucosa, attention was paid to the ultrastructural characteristics of the architecture of tumors, formation and quality of the epithelial basement membrane, junctional complexes, including hemidesmosomes, cytoplasmic structures and the size, and structure and chromatin characteristics of nuclei and nucleoli. In the assessment of our specimens, we used previously described data of ultrastructural estimation [31–33]. In our study, special attention was paid to the characteristics of cell nuclei. Polymorphism of these structures includes differences in size, nuclear envelope contour (regular, oval without deep invagination, to deep indentation into the chromatin to polykaryocytes, with a lack of nuclear envelope on some small areas of nuclei), and structure of chromatin (heterochromatin beneath the nuclear envelope to highly euchromatic nuclei with prominent and also multiple, nucleoli). Additionally, in some tumor areas, regular as well as irregular mitotic figures were present. In our study group, there were statistically significant correlations between nuclei polymorphism of A and B with T stage (*p* < 0.0001), N stage (*p* < 0.046), and metastases to the lymph nodes confirmed in histopathological examination (pN *p* < 0.004). At the same time, the second examined parameter, i.e., the number of cells in irregular mitosis for the same area, correlated with the T stage (*p* < 0.004) and highly with pN (*p* < 0.009). These findings could explain the dynamics of cancer biology and could also be very useful as a prognostic factor in oral cancer.

We also analyzed ultrastructure of the resection margin because data regarding the prognostic importance of HE-stained specimens are not clear-cut. Some authors conclude that it is a robust prognosticator, whereas others contradict this thesis [34–39]. Going to the details, the first group supports the thesis that the presence of single cell or groups of cells in stroma surrounding the main tumor texture in the resection margin is a separate, negative predicting factor. In the present study, this situation did not correlate with worse prognosis. This may be at least partly explained by the fact that patients after surgery routinely undergo additional therapy, i.e., irradiation and/or chemotherapy which may be sufficient to eliminate cancer cells and prevent local relapse.

The ultrastructural characteristics of cell nuclei, including their heterogeneity encouraged us to confront the morphologic data with epigenetic profile. Solid tumors, like many other human diseases, show aberrant epigenetic alterations [19, 40–42]. Methylation of CpG islands associated with tumor suppressor genes is found to be crucial in the multistep mechanism of carcinogenesis. In our study, gene hypermethylation was detected most commonly in *CDH1* and *CDKN2A* genes in central and peripheral parts of the tumor. These alterations prevailed in the peripheral part of the tumor, what may be associated with the presence of more rapidly proliferating cells that are more susceptible to molecular changes. However, only one statistically significant correlation was established between *CDH1* in the B zone and N stage. Data regarding the hypermethylation process and its influence on clinicopathological features and prognosis are still unclear. Ha and Califano [18] noticed that methylation of *CDKN2A* ranged from 0 to 85 % in oral cancer. Nagata et al. found that methylation of four statistically selected genes including *E-cadherin* and *RAR-*β allows to detect oral cancer with 100 % sensitivity and 87.5 % specificity [43].

In our study, *CDKN2A* hypermethylation did not correlate with clinicopathological data, except survival, which was observed in the Kaplan–Meier curve. Hence, our observations for only this one parameter are in agreement with other authors, claiming the usefulness of the determination of the methylation status of *CDKN2A* as prognosis marker [44–47]. Dong et al. [44] found a statistically significant correlation between methylation of *CDKN2A* and nodal involvement and survival in buccal carcinoma, however, without association with gender, age, T stage, and G stage. In this study, the overall survival was poorer for the group with hypermethylation in the Kaplan–Meier test, but in multivariable analysis using the Cox regression model methylation was not statistically correlated with survival. In a multicentre study covering 353 cases of head and neck cancer, Roh et al. found that *CDKN2A* methylation correlated with a decreased survival [45]. However, in tumors with disruptive *TP53* mutation, methylation of *CDKN2A* was protective. In our study, *TP53* mutations were not tested. Veganzones-de-Castro et al. working on colorectal cancer made the same observation as we did [48]. They found that patients with poorly differentiated tumors having *CDKN2A* promoter methylated presented longer free survival than those without *CDKN2A* methylation. We cannot rule out that only one allele was methylated and the other one still remained and performed its function.

The methylation of *RAR-*β and *FHIT* was found less frequently in the central and peripheral parts of the tumor. In our study, methylation of these two genes did not correlate with age, stage of the tumor, malignancy grading, or survival. However, the methylation in tumor zone was more prevalent than in the resection margin. Tanaka et al. [49] found that methylation of the 5′ CpG island is an important mechanism for the inactivation of the *FHIT* gene in esophageal carcinoma. Hypermethylation of *FHIT* was relatively often found in oral cancer (27 %) [18] and in laryngeal carcinoma (26 %) [50]. However, it did not correlate with clinicopathological data and survival. *RAR-*β is completely suppressed in immortal dysplasia and oral carcinoma [24]. Hypermethylation was often found in oral cancer (47–100 %) [18], and relatively often in the laryngeal cell lines (64.7 %) [51]. Paluszczak et al. found the methylation of this gene in 59 % of laryngeal carcinoma cases, but it did not correlate with prognosis [50]. We found the same statistical results in our study. In our group of patients, we did not find aberrant methylation of *ATM*. Hypermethylation of this gene in HNSCC was described as connected with poorer prognosis [23, 52] but we did not confirm these observations.

The molecular assessment of the surgical margin was an important part of our research. In our study, we did not find statistically significant correlation between methylation of the resection margin and prognosis. Methylation of *CDKN2A* in the surgical margin was found as a prognostic factor for tongue cancer by Sinha et al. [20]. Other authors, however, found molecular changes in the resection margin, but without correlation with survival [21, 53]. Some authors propose criteria which determine the reliability of a molecular marker in the resection field [54, 55]. After the precise analysis Bradley et al. [54] concluded that the *TP53* mutations fulfil the proposed criteria. Finally, we should keep in mind that cancer recurrence could have many reasons, which was precisely described by Braakhuis et al. [56].

In conclusion, we would like to stress that ultrastructural assessment in our patient group brought important information about cancer biology. Parameters like nuclei polymorphism and number of cells in irregular mitosis are strong prognosticators of patient survival. The epigenetic changes found in the tumor area did not correlate with clinicopathological features except *CDKN2A* methylation, which was correlated with better prognosis. The molecular assessment of the resection margin did not bring new prognostic information. However, a longer observation time and a larger number of patients are required to draw final conclusions.

Overall, we report frequent changes in tissue ultrastructure and gene hypermethylation of *CDKN2A* and *CDH1* in oral cancer. The inclusion to our study of the estimation of other molecular parameters may result in the discovery of novel, prognostic markers of this disease.
